# Contemporary Review of Multi-Modality Cardiac Imaging Evaluation of Infective Endocarditis

**DOI:** 10.3390/life13030639

**Published:** 2023-02-25

**Authors:** Aro Daniela Arockiam, Ankit Agrawal, Joseph El Dahdah, Bianca Honnekeri, Tahir S. Kafil, Saleem Halablab, Brian P. Griffin, Tom Kai Ming Wang

**Affiliations:** Section of Cardiovascular Imaging, Department of Cardiovascular Medicine, Heart, Vascular and Thoracic Institute, Cleveland Clinic, Cleveland, OH 44195, USA

**Keywords:** infective endocarditis, multi-modality imaging, echocardiography, transesophageal echocardiography, computed tomography, nuclear imaging, positron emission tomography, magnetic resonance imaging

## Abstract

Infective endocarditis (IE) remains to be a heterogeneous disease with high morbidity and mortality rates, which can affect native valves, prosthetic valves, and intra-cardiac devices, in addition to causing systemic complications. The combination of clinical, laboratory, and cardiac imaging evaluation is critical for early diagnosis and risk stratification of IE. This can facilitate timely medical and surgical management to improve patient outcomes. Key imaging findings for IE include vegetations, valve perforation, prosthetic valve dehiscence, pseudoaneurysms, abscesses, and fistulae. Transthoracic echocardiography continues to be the first-line imaging modality of choice, while transesophageal echocardiography subsequently provides an improved structural assessment and characterization of lesions to facilitate management decision in IE. Recent advances in other imaging modalities, especially cardiac computed tomography and ^18^F-fluorodeox-yglucose positron emission tomography, and to a lesser extent cardiac magnetic resonance imaging and other nuclear imaging techniques, have demonstrated important roles in providing complementary IE diagnostic and prognostic information. This review aims to discuss the individual and integrated utilities of contemporary multi-modality cardiac imaging for the assessment and treatment guidance of IE.

## 1. Introduction

**Figure 1:**
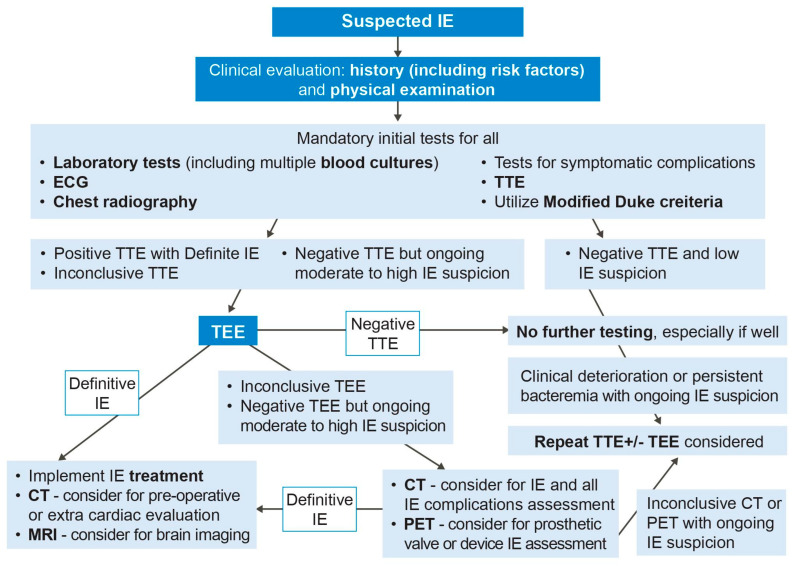
Multi-modality imaging diagnostic algorithm for infective endocarditis. IE = infective endocarditis, TTE = transthoracic echocardiography, TEE = transesophageal echocardiography, CT = computed tomography, MRI = magnetic resonance imaging, PET = positron emission tomography.

Infective endocarditis (IE) refers to an infection of the endocardial surface structures of the heart [1,2]. It is a complex heterogeneous condition often with systemic complications and carries a high rate of mortality and morbidities. The diagnosis of IE is traditionally based on the modified Duke criteria (Table 1), but remains challenging in many clinical scenarios, and delayed diagnosis may lead to irreversible harm to patients [3]. Transthoracic echocardiography (TTE) is the first-line imaging modality for assessing IE, and transesophageal echocardiography (TEE) is required in the vast majority. Computed tomography (CT), nuclear imaging such as ^18^F-fluorodeoxyglucose positron emission tomography/CT (^18^F-FDG-PET/CT) and cardiac magnetic resonance imaging (CMR) are increasingly utilized as complimentary cardiovascular imaging techniques for identifying IE and its complications, with several niche indications. Together, multi-modality cardiac imaging plays a critical role in the evaluation and treatment guidance of IE towards medical and surgical therapies. In this review, we aim to discuss the various multi-modality imaging techniques that are used for early prompt diagnosis and management of IE. Our proposed IE diagnostic algorithm using multi-modality imaging is summarized in Figure 1.

## 2. Clinical Perspectives

### 2.1. Presentation

IE can be thought of as developing on native heart valves, prosthetic heart valves, or intracardiac implantable electronic device components and is most commonly identified as vegetations macroscopically in cardiac imaging [2]. Disruption to the valvular endothelial surface or inflammation creates a nidus for pathogens where they adhere to the surface. Activated endothelial cells commence the von Willebrand factor deposition and subsequent platelet and fibrin deposition form thrombotic vegetation, promoting bacterial growth [4]. IE can affect both the left- and right-sided heart valves, which have different clinical presentations. Beyond vegetations, IE has several other intracardiac manifestations, such as abscess, pseudoaneurysm, valve perforation, prosthetic valve dehiscence, and fistulas, and together these can lead to significant valvular dysfunction (usually regurgitation), heart failure, heart block, and sepsis [2,5]. Systemic complications are also confirmed as a result of septic embolism from IE, such as the affecting of the lungs (from right-sided IE), or the brain, abdominal organs, and limbs (left-sided IE). The onset of IE can be divided into acute and subacute [6,7]. Acute infection is a sudden onset with clinical presentation of sepsis and septic shock and multi-system metastasis. Subacute infection has insidious onset and presents with nonspecific symptoms, such as fever, chills, sweats, or shortness of breath. Other clinical signs include skin lesions such as Osler’s nodes, Janeway lesions, splinter hemorrhages, petechiae, and clubbing [8].

### 2.2. Risk Factors

Understanding the risk factors of IE is important in its evaluation and management. Prosthetic valves, cardiac implantable electronic devices, and prior endocarditis are major risk factors that raise clinical suspicion. Several of these are high risk groups that may warrant antibiotic prophylaxis discussed below [1,2]. Other important risk factors include older age, male sex, structural heart disease including mitral valve prolapse, bicuspid aortic valve, rheumatic heart disease and congenital heart disease, heart transplantation, intravenous drug use (which is an epidemic in some countries that has increased the incidence of right-sided IE), dental infections, hemodialysis or other use of indwelling catheters, and immunosuppressive states such as HIV [9,10].

### 2.3. Microbiology

The most common causative microorganism in IE used to be the Streptococci viridans species. Recent epidemiological studies have seen the rise of Staphylococcus aureus to being known as the most common in developed countries, likely associated with healthcare contact and procedures including catheters and prosthetic valves, along with intravenous drug use, and this pathogen often has a more aggressive disease course. The other main IE pathogens include Enterococcus, coagulase negative Staphylococci (especially for prosthetic valves), HACEK organisms (*Haemophilus parainfluenza*, *Actinobacillusactinomycetemcomitans*, *Cardiobacterium hominis*, *Eikenella* species, *Kingella* species), and less commonly other bacteria such as the Bartonella, Mycoplasma, Legionella, and Brucella species and fungal infections such as Candida [11]. Identifying bacteremia based on blood cultures taken multiple times from multiple sites is an important first step in the diagnosis of IE, including those based on the modified Duke criteria [3]. However, culture-negative endocarditis is present in a minority of cases, whether because of preceding antimicrobial therapy, or the causative microorganism being difficult to grow or intracellular so that it cannot be detected via culturing blood alone. Serology and polymerase chain reaction (PCR) tests may help identify some of these organisms [1,2,12,13]. Valve culture and histopathology after surgery for IE may also aid in this assessment and confirming an IE diagnosis [2].

### 2.4. Management

Antimicrobial therapy remains critical for all patients with IE, and the implicated micro-organisms and risk factors (such as prosthetic valve involvement) help determine the type, number, administration, and duration of the pharmacological treatments. Early diagnosis and initiation of antimicrobial therapy may stem the progression of IE, reduce embolic risk, and improve outcomes [2]. Cardiac surgery is indicated in approximately half of all IE. Emergency surgery is required when there is severe left-sided valve regurgitation or other complications causing cardiogenic shock and refractory heart failure [1,2]. Urgent surgery is also indicated for severe valve regurgitation with heart failure, uncontrolled infection such as abscess, enlarging vegetation, fistula, fungal, or multi-resistant organisms, and the prevention of embolism, whether following evidence of embolism or sometimes very large vegetations [2]. Further discussion of microbial diagnosis and treatments of IE are outside the scope of this review.

### 2.5. Antibiotic Prophylaxis

Antibiotic prophylaxis for IE is currently recommended for dental procedures in patients with prior history of endocarditis, prosthetic heart valves, prosthetic material in valve repair (annuloplasty rings, chords, or clips), cyanotic congenital heart disease (unrepaired, repaired with residual shunt or valvular regurgitation, or within 6 months of complete repair), and heart transplant recipients with regurgitant valvulopathy [1,2]. The agent of choice is amoxicillin; however, penicillin allergy is present, then clindamycin is the next choice. Perioperative antibiotic prophylaxis is also recommended before cardiac implantable electronic device placement and should be considered for prosthetic valves and other structural heart procedures [2].

### 2.6. Endocarditis Features on Imaging

The main features of endocarditis in imaging are summarized in Table 2. Vegetations are soft tissue masses of inflammatory cells, bacteria, fibrin, and platelets which can be seen hooked to any implanted cardiac device or an endocardial surface. A pseudoaneurysm appears as an abnormal cavity, usually in the vicinity of the valve and communicating with cardiac chambers or a major blood vessel. An abscess, on the other hand, is a closed cavity with a purulent composition and with no communication with any cardiac chamber. Valve perforation refers to defects in native or prosthetic valve leaflets that allow blood to flow directly through, both in a antegrade and retrograde manner. Valve dehiscence, typically for prosthetic valves, is defined as an abnormal gap between the valve ring and the annulus it is implanted on, through which paravalvular leak flow can occur. Further, a defect in the continuity of the endocardial tissue allowing abnormal communication between the two neighboring cardiac chambers or with adjacent blood vessels and other structures is termed a fistula.

In 2015, the European Society of Cardiology (ESC) revised guidelines that highlight the importance of imaging in the management of patients with suspected IE. Indeed, new techniques are increasingly being used when TTE/TEE results are negative with a persistent high level of clinical suspicion [14]. Nuclear molecular imaging techniques provide additional value in diagnosing patients with possible IE using the modified Duke criteria. The 2017 Appropriate Use Criteria for Multimodality Imaging in Valvular Heart Disease also includes FDG-PET alongside CT, since it may be useful in suspected IE with moderate-to-high pretest probability and negative TTE. Additionally, neither the 2014 AHA guidelines nor the 2015 ESC guidelines suggest the use of molecular imaging techniques. As compared to the sensitivity for detecting PVE, the observed sensitivity for diagnosing NVE with FDG-PET was significantly low, so guidelines do not recommend it as routine first-line imaging for NVE [15]. The 2015 ESC guidelines added pseudoaneurysm, intracardiac fistula, and valve perforation or aneurysm as major imaging criteria that can be detected via echocardiography, but CT is also particularly useful in the evaluation of periannular complications of PVE [16]. However, the role of CT is very limited in CIED infections. FDG-PET, on the other hand, has become a valuable imaging technique to differentiate uncertain cases of possible lead infection. Additionally, it serves as the preferred imaging method for assessing pacemaker pocket infection, also known as local device infection [15]. FDG-PET is an effective diagnostic tool in the evaluation of challenging cases of IE, particularly prosthetic valve endocarditis [17]. Furthermore, the 2015 European Society of Cardiology modified criteria for IE diagnosis to include both CT and nuclear imaging modalities (FDG-PET and leukocyte SPECT) as major criteria when PVE is positive. However, the 2017 Appropriate Use Criteria for Multimodality Imaging in Valvular Heart Disease include that FDG-PET may be useful in detecting PVE [15].

## 3. Echocardiography

### 3.1. Transthoracic Echocardiography

Echocardiography is the cornerstone of imaging modalities used for IE assessment [18]. The modified Duke criteria, which indicate whether definitive infection or probable infection is present according to major and minor criteria (Table 1), have major echocardiographic criteria for IE diagnosis that include vegetations, abscesses, new valvular regurgitation, and prosthesis dehiscence, while other supporting features include valve perforation or aneurysm [1]. TTE is the first-line imaging test for IE and should be promptly performed as soon as IE is suspected [1,10,11,12]. A complete examination should be performed, involving all standard parasternal, apical, subcostal, and suprasternal views, and employing two-dimensional, Doppler, and three-dimensional techniques with careful interrogation on all four heart valves, including prosthetic heart valves and cardiac devices, as well as the aorta for signs of IE [19]. TTE can also assess the cardiac chamber’s size and function, as well as evaluating for congenital heart disease, pericardial conditions, adjacent vascular structures, and estimating pulmonary pressures. All echocardiography techniques excel with high temporal resolution, so they are generally better at identifying mobile vegetations, especially if they are small or thin compared to other modalities. TTE has many strengths, such as it being a first-line imaging modality, but with notable limitations, such as suboptimal sensitivity due to lower spatial resolution and prosthetic valve artifacts warranting further evaluation [11,13], as indicated in Table 3.

### 3.2. Transesophageal Echocardiography

**Figure 2:**
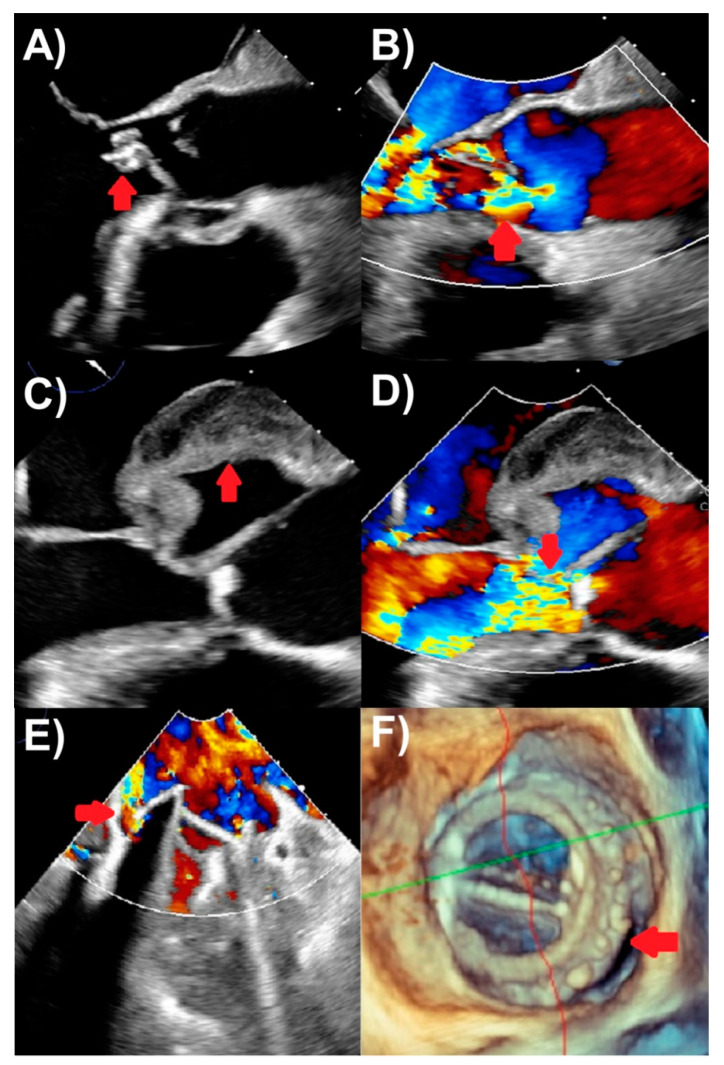
Transesophageal echocardiography findings of endocarditis. (**A**) Aortic valve vegetation (arrow), with (**B**) severe aortic regurgitation (arrow) on color Doppler. (**C**) Aortic with echolucent space consistent with pseudoaneurysm and abscess (arrow), with (**D**) severe aortic regurgitation (arrow) on color Doppler. (**E**) Mechanical mitral valve replacement paravalvular regurgitation (arrow) associated with (**F**) prosthetic valve dehiscence (arrow) seen on three-dimensional assessment.

TEE is recommended for the vast majority of IE patients in absence of contraindications, because of its superior sensitivity and specificity to TTE from high spatial and comparable high temporal resolutions [20,21]. TEE is the best modality for evaluating vegetations, valve perforation, and prosthetic valve dehiscence, also performing well to identify pseudoaneurysm, abscesses, and fistulas (Figure 2). A comprehensive TEE examination should also be performed when assessing for IE, focusing on all four heart valves in esophageal and transgastric views [19]. In particular, contemporary three-dimensional echocardiography, including multi-planar reconstruction, is critical for the accurate depiction of the presence of IE, for its etiology and features, and usually more accurate for TEE than TTE. Clinical scenarios to use TEE include when TTE is inconclusive for IE, but there is a moderate-to-high clinical suspicion for IE; TTE is negative, but there is ongoing high clinical suspicion for IE; and TTE showing features of IE, but further evaluation for complications (such as new heart murmur, high-grade heart block, suspected abscess, embolic events, and heart failure) is required [1,2]. The last of these indications makes an argument for routinely performing TEE in all patients with IE, as TTE may miss important IE complications. TEE also plays an important role in assessing patients with prosthetic valves or intracardiac devices where TTE is less accurate and more prone to artifacts. TEE is not usually required if IE suspicion is low and TTE is negative [2]. Finally, intraoperative TEE is used for patients undergoing IE surgery to assess the extent of IE complications and the surgery needed, as well as for assessing the surgical result [1]. TTE and TEE are the cornerstones of first-line imaging modalities to assess endocarditis in all IE guidelines [1,2,22].

### 3.3. Three-Dimensional Echocardiography

Three-dimensional (3D) echocardiography is now a cornerstone technique routinely performed in both TTE and TEE, including for the assessment of IE. The three-dimensional aspect has added value for many reasons in IE assessment, including when two-dimension echocardiography findings remain uncertain, since they enable more accurate anatomical delineation, localization of pathology, and measurement via multi-planar reconstruction. Real time 3D TEE permits the assessment of the 3D volumes of cardiac anatomy in infinite planes [23]. It is able to analyze vegetation size, morphological appearance, and embolic risk, and evaluate perivalvular extensions, valve perforations, and prosthetic valve dehiscence [24,25]. Furthermore, it has a valuable role especially with TEE in the intraprocedural guidance of surgical and transcatheter interventions. Limitations of 3D include dependence on the imaging quality of the 2D data and a lower spatial and temporal resolution restricting its application for small or highly mobile vegetations [26].

### 3.4. Comparisons of Echocardiography Modalities

Table 4 lists sensitivities and specificities of TTE, TEE, and other cardiac imaging modalities in identifying IE features, while stress echocardiography do not have a routine role in IE assessment. Overall, the TTE and TEE sensitivities for native valve IE were 40–60% and 90–100% respectively, and for prosthetic valve IE were 17–36% and 82–96%, respectively [18,19,20]. In particular, TTE and TEE sensitivities for vegetations were 44–69% and 87–100%, respectively, and for intracardiac abscesses 28–50% vs. 80–90%, respectively [27,28,29,30]. In patients with prosthetic valve IE, TEE is of particular interest for subaortic complications detection as they are frequently underestimated with TTE [31]. Detection of abscesses or pseudoaneurysms in patients via TEE is also independently associated with increased in-hospital mortality and morbidity [32]. TEE also is the preferred modality to assess paravalvular leaks and has a higher accuracy than TTE for identifying leaflet perforation, prosthetic valve dehiscence, and fistulas. For perforation, the sensitivity and specificity of TEE was reported to be 79% and 93%, respectively [33]. Additionally, the sensitivities and specificities for abscess, fistulas, and dehiscence were 70.3% vs. 95.5%, 85.7% vs. 98.6%, and 66.6% vs. 99.2%, respectively [33]. Furthermore, echocardiography can also predict the embolic risk associated with IE lesions. Although embolic risk is multifactorial, two of the strongest predictors are the size and mobility of the vegetations [34,35,36,37,38]. Neurological emboli are seen most in large vegetations (>3 cm in greatest dimension) [39].

### 3.5. Repeating Echocardiography

In some clinical scenarios, repeat TTE and/or TEE should be considered. In events where both TTE and TEE assessments are negative but clinical suspicion of IE remains high, another TEE should be scheduled. This is especially true when new IE complications arise after initial echocardiographic assessment, such as embolism, heart failure, abscess, a new murmur, atrio-ventricular block, or when there is persistent evidence of sepsis and bacteremia for at least one week despite appropriate antimicrobial treatments [45]. However, no clear consensus exists on the optimal timing of repetition since it greatly depends on the patient’s pathology and risk status. The ACC/AHA 2020 guidelines recommend a TEE repetition 3–5 days after first TEE evaluation, while the ESC guidelines recommend 7–10 days wait before repetition [1,2]. Some studies suggest less of a waiting time before echocardiography repetition in higher risk patients such as those with suspected prosthetic valve IE or Staphylococcus aureus infection [38,46]. This short-term interval repetition should enhance the sensitivity of the assessment [43], although an alternative approach is pursuing other complimentary imaging modalities. Echocardiography should also be repeated at the end of an antimicrobial course to assess for the improvement and resolution of IE findings, and for routine surveillance after valve surgery.

## 4. Cardiac Computed Tomography

### 4.1. CT Techniques

A dedicated cardiac protocol with iodinated contrast administration using multi-detector CT with at least 64-slice technology is necessary for accurate IE evaluation [47]. The preference is to utilize the four-dimensional CT technology using retrospective ECG gating to acquire images after contrast administration in the arterial phase of the heart throughout the cardiac cycle (10–20 phases every 5–10% of the RR interval), with the thin slices typically 0.60–0.75 mm for optimal spatial resolution and analyzed using dedicated software with multiplanar reconstruction. The latter is critical to reconstruct views of all cardiac structures in any plane for evaluation. A subsequent ECG-gated imaging of the entire thorax, and if necessary, abdomen, pelvis, and head; these reasons may be considered depending on if there are concerns about systemic embolism. ECG gating, treating (such as with beta-blockers) patients with arrhythmias, and encouraging breath hold acquisition can assist in reducing cardiac and respiratory motion artifact. Strengths and limitations of CT is indicated in Table 3, the limitations including radiation exposure, which is especially higher with four-dimensional imaging, the caution required in renally impaired and iodine allergies patients with contrast administration, and inferior temporal resolution to echocardiography [44,48]. The radiation dose may be reduced by using a lower tube voltage, ECG-controlled current modulation, while a lower contrast dose may sometimes be necessary in those with chronic kidney disease.

### 4.2. CT Evaluation of IE

**Figure 3:**
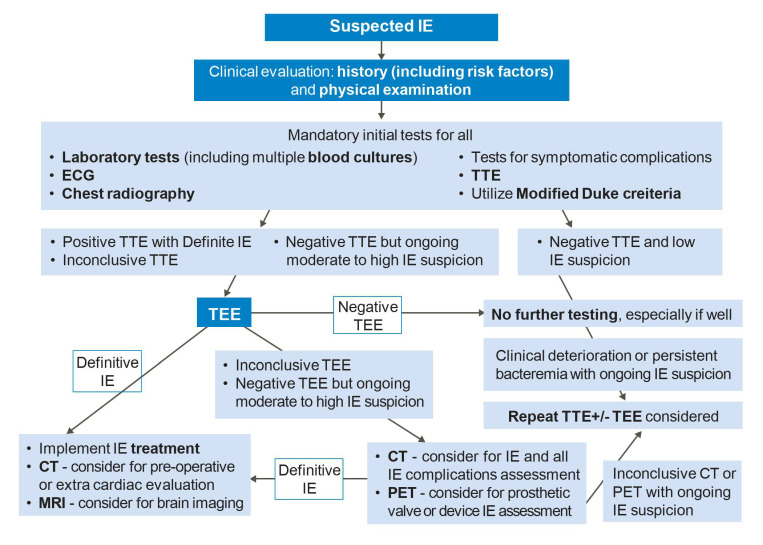
Cardiac computed tomography with retrospective ECG gating and iodinated contrast examples of infective endocarditis manifestations: (**A**) bioprosthetic aortic valve vegetations (white arrows), (**B**) aortic root pseudoaneurysm with fistula connection to the left ventricular outflow tract (yellow arrow), with vegetations (white arrow), (**C**) aortic root abscess (yellow arrows) and bioprosthetic aortic valve thickening (white arrow may represent endocarditis, thrombus, or pannus depending on clinical setting), and (**D**) bioprosthetic aortic valve dehiscence with aortic root pseudoaneurysm (yellow arrow).

CT had become a critical imaging modality for evaluation in IE, complimentary to TTE and TEE. It is utilized for cardiac evaluation if the diagnosis of IE remains inconclusive but there is a moderate-to-high clinical suspicion following TTE and TEE (or if TEE is contraindicated) as well as for assessing IE intracardiac complications that are not well visualized on echocardiography (Figure 3) [1,2]. Sensitivities and specificities for CT performance at detecting endocarditis features are shown in Table 4.

Vegetations are seen as hypodense homogenous irregular masses attached to a valve or other cardiac anatomical structures. CT has moderately high sensitivity and specificity for vegetations, the former being slightly lower than TEE due to its inferior temporal resolution to echocardiography to detect smaller sub-centimeter or very mobile vegetitations [33]. It should also be noted that sometimes IE can be seen as only as a valve leaflet thickening without other abnormalities in a CT scan, which can be considered a non-specific finding that should be interpreted with the clinical context in mind.

A main role of CT is its high sensitivity and specificity for periannular complications such as pseudoaneurysms and abscesses, with a trend towards a higher sensitivity than TEE because of the great spatial resolution of CT [20,33]. This is true for both native and prosthetic valve IE and is an important adjunctive role for CT when such findings remain uncertain on TEE. Furthermore, CT also has a higher accuracy for identifying fistulae associated with IE, and moderate-to-high accuracy for identifying paravalvular leaks [33].

On the other hand, CT has a lower sensitivity of just under 50% for identifying other valvular abnormalities in IE, in particular native valve leaflet perforation, along with prosthetic valve dehiscence, although the specificity remains high [33]. In both cases, TEE is the preferred modality for assessment rather than CT because of the higher temporal resolution and color Doppler techniques of echocardiography.

Beyond diagnosis, CT also has important prognostic roles in IE evaluation. In a recent study of 123 patients with IE, CT detection of pseudoaneurysms or abscesses and CT detection of fistulas were the only dependent predictors of a higher mortality risk during follow-up, with hazards ratios of 3.8 and 9.8, respectively [32].

Current guidelines endorse the utility of CT in endocarditis evaluation. The 2015 European Society of Cardiology guidelines include CT findings as major criteria, especially for prosthetic valve IE [2]. The 2017 Appropriate Use Criteria for Imaging in Valvular Heart disease mentions that CT may be appropriate in suspected IE with moderate-to-high pretest probability after a negative TTE, however not when used as an initial evaluation modality [22]. The 2020 American College of Cardiology and American Heart Association Valvular Heart Disease guidelines emphasize CT’s role in evaluating prosthetic valve IE and other IE complications, including right-sided IE, pulmonary infarcts, and abscesses along with its role in performing pre-operative evaluations that include coronaries and aortic involvement [1].

### 4.3. Other Roles of CT in IE

CT has several other roles in evaluating IE beyond the heart itself. It is a useful imaging tool for the pre-operative planning of cardiac surgery, to evaluate the anatomical locations and relations of the sternum, great arteries, veins, and heart, and is especially important for redo sternotomies that are applicable to prosthetic valve IE. If the only role for CT is pre-operative planning, then a single-phase, ECG-gated CT chest protocol with or without contrast is sufficient. The thoracic aorta size and IE involvement may impact on the need for concomitant aortic surgery. Coronary artery anatomy and disease can be assessed in CT by a using thin slice contrast-enhanced ECG-gated sequence (which the aforementioned four-dimensional IE protocol can also achieve), which may also be useful as a rule-out tool and when invasive catheterization is risky in the presence of aortic valve vegetations and root abscesses. However, if there is more than moderate disease or more atherosclerotic disease and stenosis, then invasive coronary catheterization is typically performed for precise delineation and assessing the need for revascularization [49]. The four-dimensional acquisition also allows for the quantification of the cardiac chamber size and ejection fraction. The other important role for CT is the assessment for embolic events, present in 20–50% of the IE patients [37], and a minor diagnostic criterion for IE [3]. The CT body or dedicated CT scans to the region of interest may identify complications such as brain lesions, including ischemic stroke, cerebral abscesses, and intracranial bleeding; pulmonary septic emboli; vascular defects such as mycotic aneurysms; abdominal visceral infarcts in the kidney, spleen, and bowels; musculoskeletal defects such as osteomyelitis and septic arthritis; and peripheral limb ischemia.

## 5. Cardiac Magnetic Resonance Imaging

CMR has a relatively limited role in IE evaluation, with advantages and limitations seen in Table 3. CMR has become the reference standard for chamber size and function quantification and can also assess and quantify valvular heart disease, especially for valve regurgitation, which is common in IE [50,51]. CMR can also uniquely perform tissue characterization, including using late gadolinium enhancement imaging to detect myocardial and pericardial inflammation, as well as parametric mapping techniques [52]. Despite the excellent spatial resolution, CMR has a lower temporal resolution than echocardiography, and often misses smaller vegetations. CMR is also challenged by artifacts associated with prosthetic valves and device leads as well as non-CMR conditional items [53,54]. The role of magnetic resonance imaging in IE extends beyond intracardiac manifestations. Magnetic resonance imaging (MRI) of the brain can provide accurate information of any cerebrovascular event in the case of vegetation embolization [53,55]. Magnetic resonance angiography is also an alternative to computed tomography angiography for assessing mycotic aneurysms, with pooled sensitivities and specificities of 79% and 89%, respectively, in one meta-analysis [56]. Indeed, guidelines have refrained from recommending CMR in IE evaluation, except for MRI of the brain for cerebral complications and its angiography for mycotic aneurysm evaluation [2,22].

## 6. Nuclear Imaging

Nuclear imaging modalities have rapidly evolved over the last two decades and are increasingly utilized for IE evaluation, especially ^18^F-FDG-PET/CT and radiolabeled leukocyte white blood cell scintigraphy (WBC SPECT/CT). Nuclear imaging detects inflammatory and functional changes which often develop prior to anatomical changes detected on other imaging modalities, and thus if performed early, may improve outcomes due to earlier initiation of treatment [15,57,58]. As per American Heart Association/American College of Cardiology 2020 guidelines, ^18^F-FDG-PET can be a helpful major criterion in the modified Duke criteria, especially for prosthetic valve endocarditis [1]. Both ^18^F-FDG-PET/CT and WBC-SPECT/CT nuclear imaging modalities provide added value in assessing for extracardiac lesions including septic emboli and extra-cardiac pockets of infection.

### 6.1. ^18^F-FDG PET/CT

^18^F-FDG-PET/CT is able detect inflammatory functional changes in advance of the development of morphological changes, enabling for an early diagnosis of IE, which may prompt early initiation of treatment and thus improve outcomes [34,57]. It is generally utilized if diagnosis remains limited and uncertain after TTE and TEE, or if TEE is contraindicated. A recent meta-analysis pooled data from 26 studies of ^18^F-FDG-PET/CT for IE [44]. They identified very high specificity for all IE, but the sensitivity for diagnosis was much lower at <50% in most studies for native vale IE (Table 4), so its role is relatively minor in native valve IE, although the high specificity may be occasionally useful [17,29,48,59].

**Figure 4:**
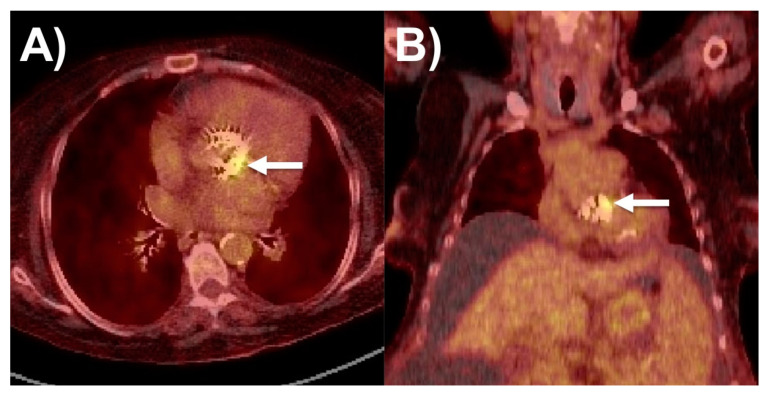
18-Fludeoxyglucose positron emission tomography case of prosthetic aortic valve endocarditis with radiotracer uptake (arrow) on (**A**) axial and (**B**) coronal views.

^18^F-FDG-PET/CT has a more robust role in the evaluation of prosthetic valve endocarditis (PVE), where it had both a higher pooled sensitivity (86%) than for NVE as well as a high pooled specificity (84%), such as in Figure 4. More recent trials show higher sensitivity and specificity than older ones. It has comparable performance in both biological and mechanical prosthetic valves [15,17,59,60,61,62,63]. It is especially useful when prosthetic valve-related artefacts impair assessment via TTE and TEE [64,65]. It is also useful in the setting of culture-negative IE with fastidious organisms [44,57,59,60,61,62,63]. From a prognostic standpoint, positive FDG valvular uptake is associated with worse outcomes (death, hospitalizations, IE recurrence, acute heart failure, new embolic event) [66].

For cardiac device-related infective endocarditis (CDRIE), ^18^F-FDG-PET/CT has poor sensitivity (65%) for lead infections and thus mainly has a role in evaluating inconclusive cases. It has much better sensitivity (93%) for pocket infections, making it an imaging modality of choice. It has high specificity for both forms of CDRIEs (98% and 88% respectively) [57,63,67]. There may also be prognostic value for ^18^F-FDG-PET/CT prior to lead extraction in confirmed lead CDRIE without pocket involvement, as these patients were found to have worse outcomes after lead extraction. Similar to NVE and PVE, ^18^F-FDG-PET/CT is key for detecting septic emboli originating from cardiovascular devices, with several studies showing detection of extracardiac foci in about 20% of patients [57,68,69,70]. In recent, smaller studies, ^18^F-FDG-PET/CT has also been demonstrated to have high pooled sensitivity (97%) and specificity (93–99%) for LVAD infections [57,71]. LVAD infections are diagnostically challenging and difficult to treat, as source culture and source control (device removal) may not be easily possible. Similarly, it may also have a key role in diagnosing vascular graft infection (VGI)—both native vessel and endoprosthesis—for which it has high pooled sensitivity (>90%) and moderate-to-high pooled specificity (59–81%) [72,73,74,75].

^18^F-FDG-PET/CT is especially of value in the detection of extracardiac foci (disseminated disease, septic emboli, portals of entry), which can establish diagnosis when clinical findings and echocardiography are not sufficiently revealing [57,76]. Detection of septic emboli fulfils a minor criterion in the modified Duke criteria, thus the addition of ^18^F-FDG-PET/CT improves the sensitivity of the criteria for NVE without impacting its high specificity [57,76]. It could also be used to evaluate the extent of disease burden [77]. Notably, moderate to high valvular FDG uptake was associated with more septic embolic phenomena [66].

^18^F-FDG-PET/CT has a limited role in the post-operative setting. Sterile inflammatory changes in the 3 months after surgery may be indistinguishable from infection. Further, interpretation can be challenging if there is focal FDG uptake in the presence of concomitant conditions such as vasculitis, active thrombus, or malignancy. While it helps detect peripheral embolic events in the body, physiologically high ^18^F-FDG uptake in the brain precludes its use for cerebral septic embolism [15]. Antibiotic administration prior to imaging increases the false negative rate, and this appears to be related to duration of therapy [57,77,78,79]. Early nuclear imaging is especially of relevance, as detection of septic emboli often changes the course of management (prolongation of antibiotic therapy, early consideration of surgery), and earlier initiation of antibiotic therapy is associated with improved outcomes.

Hybrid ^18^F-FDG-PET combined with CTA is an imaging modality that also allows for the simultaneous evaluation of the coronary arteries prior to surgical consideration. It is finding increasing relevance in TAVI-associated IE, where echocardiography images are often obscured by metal artefacts [57,78,80,81,82]. ^18^F-FDG-PET/CTA performs better than nonenhanced CT in detecting IE and may help differentiate infections from inflammatory perivalvular FDG uptake in PVE [57,83,84,85].

Recent guidelines have increasingly recommended nuclear imaging’s evolving role in IE assessment. The 2015 European Society of Cardiology guidelines include ^18^F-FDG-PET/CT and SPECT/CT as major criteria, especially for prosthetic valve IE [2]. The 2017 Appropriate Use Criteria for Imaging in Valvular Heart disease also states that ^18^F-FDG-PET/CT, but not SPECT/CT, may be appropriate in suspected IE with moderate-to-high pretest probability after a negative TTE, and neither are recommended for initial evaluation [22]. The 2020 American College of Cardiology and American Heart Association Valvular Heart Disease guidelines also support ^18^F-FDG-PET/CT for prosthetic valve IE, including as a criterion to improve the diagnostic capability of modified Duke criteria, and maybe a complementary tool for native valve IE; however, the latter should be cautioned due to its low sensitive in this setting [1].

### 6.2. WBC SPECT/CT

Radiolabeled leukocyte scintigraphy (WBC SPECT/CT) is another nuclear imaging modality that can be complementary in establishing the diagnosis of IE [57,86,87]. It has demonstrated high specificity early (<3 months) after prosthetic valve or device insertion, when ^18^F-FDG-PET has limited diagnostic ability [88]. It offers the advantage of intracardiac and extracardiac evaluations in a single study and is clinically useful for diagnosing septic emboli. Several small studies have established that it has a high specificity (85–100%) for IE (both NVE and PVE). Adding WBC-SPECT to the modified Duke–Li score correctly reclassified 25% of patients from possible to definite PVE. WBC-SPECT/CT should be performed as early as possible, as antibiotic therapy can lead to false negative results. The ^99m^Tc-WBC uptake also depends on the type of infection. Uptake is higher in abscesses and lower in non-abscessed lesions. It may have prognostic value, as high uptake is associated with poorer outcomes [15,57,65].

WBC-SPECT/CT in CDRIE and/or in LVAD-IE was found to have a diagnostic sensitivity of 60–93.7% and specificity of 81–100% [15,57,79,89,90]. WBC-SPECT/CT is especially of value in cases classified as possible based on the Duke–Li criteria. The addition of WBC-SPECT to the Duke criteria improved the diagnostic accuracy from 83% to 88% [58,89,90,91]. It also has a prognostic role in CDRIE, where positive WBC scintigraphy has been associated with higher in-hospital mortality, complication rate, and frequency of hardware removal [91]. Studies of WBC-SPECT/CT in LVAD-IE and vascular graft infection (VGI) have revealed a comparable performance to CRIE. It provides a diagnostic value even when performed within the first month post-operatively [15,57,79,89,90].

## 7. Future Directions of Multimodality Imaging

Multimodality imaging for IE evaluation continues to develop and evolve with the advancing field of medicine and technology. Echocardiography techniques have improved towards the higher spatial, temporal, and three-dimensional resolution of pathology to better depict endocarditis features and complications. Multi-detector CT scanners are increasingly utilized and available with higher spatial resolution and less radiation exposure. CMR techniques have advanced to also increase the spatial and temporal resolution, allowing for free breathing, the utilization of four-dimensional flow sequences, while also decreasing the scan acquisition time. Combined PET/CMR can help characterize the vegetation and anatomy at the same time [15]. Bacteria-specific tracers which are solely metabolized by bacteria and antibody tracers against the bacterial cell membrane are being studied as a part of advanced molecular and nuclear imaging and allow for discrimination between infectious and inflammatory etiologies [92]. Fusion imaging may improve the spatial delineation and functional assessment of endocarditis findings. Lastly, machine learning has ever-increasing roles in improving the efficiency of both scan acquisition and interpretation for now and the future.

## 8. Conclusions

Despite recent advances in diagnosis and management, IE continues to have high rates of adverse outcomes. Accurate and timely diagnosis is therefore critical for the early implementation of appropriate medical and surgical treatments. In addition to microbial identification, multimodality imaging is critical in the diagnostic and prognostic evaluation of IE. Transthoracic and transesophageal echocardiography are first-line and mandatory. CT and FDG-PET/CT have important complementary roles, especially when the diagnosis remains uncertain after TTE and TEE, when TEE is contraindicated, and especially in prosthetic valve and CIED endocarditis, with lesser roles in native valve endocarditis. As discussed, each modality has their strengths and limitations in characterizing IE features such as vegetations, abscesses, pseudoaneurysm, valve perforation, dehiscence, and fistulae, and working together by using our proposed algorithm can aid in IE management to improve patient outcomes.

## Figures and Tables

**Table 1:** Diagnosis of infective endocarditis based on the modified Duke criteria [3].

**Major Criteria**	
**I. Positive blood cultures for IE**	
a.	Two separate blood cultures with typical microorganism consistent with infective endocarditis, in the absence of a primary focus (viridans streptococci, Streptococcus bovis, Staphylococcus aureus, HACEK group or a community-acquired enterococci).
b.	Microorganisms consistent with IE from persistently positive blood cultures defined as follows: at least 2 positive cultures of blood samples drawn >12 h apart; or all of 3 or a majority of ≥4 separate cultures of blood (with first and last sample drawn at least 1 h apart).
c.	Single positive blood culture for Coxiella burnetii or antiphase I IgG antibody titer > 1:800.
**II. Evidence of endocardial involvement**	
	Positive echocardiogram for IE, i.e., vegetation, abscess, new partial dehiscence of prosthetic valve, new valvular regurgitation (worsening, changing, or preexisting murmur is not sufficient).
**Minor Criteria**	
1	Predisposition, predisposing heart condition, or history of drug injection.
2	Fever, temperature >38 °C or >100.4 °F.
3	Vascular phenomena, major arterial emboli, septic pulmonary infarcts, mycotic aneurysm, intracranial hemorrhage, conjunctival hemorrhages, and Janeway’s lesions, petechiae, or purpura
4	immunologic phenomena such as: glomerulonephritis, Osler’s nodes, Roth’s spots, and rheumatoid factors.
5	Microbiological evidence not fitting major criteria: positive blood culture but does not meet a major criterion as noted above or serological evidence of active infection with organism consistent with IE.
Definite IE	2 major criteria or, 1 major + 3 minor criteria or, 5 minor criteria
Possible IE	1 major criterion + 1 minor criterion or, 3 minor criteria

Notes—HACEK = *Haemophilus* species, *Aggregatibacter* species, *Cardiobacterium hominis*, *Eikenella corrodens*, and *Kingella* species.

**Table 2:** Characteristic imaging features of infective endocarditis.

Diagnostic Findings	Echo Findings	MDCT Characteristics
Vegetation	Intracardiac oscillating or non-oscillating mass seen attached to valvular surface, chamber walls, or any intracardiac device.	Hypodense, homogenous, irregular with low-to-intermediate attenuation mass attached to the endocardial surface, native or prosthetic valve, or any other cardiac implantable device.
Abscess	Irregular shaped, perivalvular, non-homogenous mass.	Perivalvular collection of a low attenuated area with peripheral contrast enhancement.
Pseudoaneurysm	Pulsatile perivalvular space which is echo free with communication with cardiac chambers.	Contrast-filled cavity in the perivalvular area with a direct communication with cardiac chamber.
Leaflet perforation	Defect in the leaflets with flow through the defect.	Defect in continuity of the valve leaflets.
Prosthetic valve dehiscence	Paravalvular regurgitation with or without rocking motion of the prosthetic device.	Presence of contrast material in the tissue defect between the annulus and the misaligned prosthetic valve.
Fistula	Connection between two adjacent intracardiac cavities.	Contrast material-filled tract between two cardiac chambers.
Aneurysm	Leaflets with saccular outpouchings with embarrassment of the leaflet curvature.	Leaflet outpouching.
Paravalvular leak	Abnormal Doppler flow in the perivalvular region.	Distinctive presence of the contrast agent column in the periannular region.

**Table 3:** Strengths and limitations of multi-modality imaging for evaluating infective endocarditis.

Imaging Modality	Strengths	Limitations
Transthoracic echocardiography (TTE)	Widely available, first-line modality, safe with no radiation exposure, portable, high temporal resolution, assesses hemodynamics, valve function, endocarditis features, and chamber function.	Operator and patient dependent on imaging windows, creates artifacts, lower sensitivity than more advanced modalities in identifying most endocarditis features including small vegetations, periannular complications, prosthetic valve, and device-related endocarditis.
Transesophageal echocardiography (TEE)	Portable, higher sensitivity than TTE for most endocarditis features, preferred modality for vegetations, valve perforation, prosthetic valve dehiscence and paravalvular leak, identifies fistula, high spatial and temporal resolution.	Invasive imaging modality, may still have artifact and lower sensitivity for some prosthetic valves and cardiac devices, avoid in contraindications such as prior gastroesophageal disease and surgery, active bleeding, patient intolerance.
Cardiac CT	Short study, excellent for detection of perivalvular complications (pseudoaneurysm, abscess, and fistula) in all types of endocarditis, can also identify other endocarditis features, detect extracardiac complications, high spatial resolution, use for pre-operative workup, and assesses coronaries and major vessels.	Non-portable, lower sensitivity than echocardiography for smaller vegetations, perforations, and paravalvular leaks. Inferior temporal resolution to echocardiography, radiation exposure, iodinated contrast administration (avoid in chronic renal impairment, especially when creatinine clearance below 30).
Cardiac Magnetic Resonance	Can identify endocarditis complications in some scenarios, such as using its high sensitivity for cerebral lesions. Reference standard for chamber quantification and can also quantify valve disease and shunts (such as for fistula).	Long study, non-portable, can cause claustrophobia, cost, non-compatible devices, lower temporal resolution than echocardiography, only for stable patients who can lie flat and follow instructions.
^18^F-fluorodeoxyglucose positron emission tomography/computed tomography (^18^F-FDG PET/CT)	Improved sensitivity for prosthetic valve and device-related endocarditis in some scenarios.	Non-portable, low sensitivity for native valve endocarditis, no functional cine imaging, radiation exposure, special pre-test preparation, cost, false-positive results within 3 months after cardiac surgery, false-negative results in patients treated with antimicrobials.

**Table 4:** Sensitivities and specificities for multi-modality imaging for evaluating various infective endocarditis findings.

		All Cases		PVIE	
		Sensitivity	Specificity	Sensitivity	Specificity
Vegetations	TTE	61% [40]	94% [40]	29% [41]	100% [41]
	TEE [33]	96%	83%	89%	74%
	CCT [33]	85%	84%	78%	94%
Perivalvular complications	TTE	28% [30]	98.6% [30]	36% [41]	93% [41]
	TEE	70% [33]	96% [33]	86% [41]	98% [41]
	CCT [33]	88%	93%		
Perforation	TTE				
	TEE [33]	79%	93%		
	CCT [33]	48%	93%		
Dehiscence	TTE			11% [41]	100% [41]
	TEE	67% [33]	99% [33]	94% [41]	97% [41]
	CCT [33]	46%	97%		
All cases of endocarditis	TTE	71% [42]	80% [42]	33% [41]	100% [41]
	TEE	90% [32]	96% [32]	86% [41]	95% [41]
	CCT			~93% [43]	95% [43]
	PET [44]	74%	88%	86%	84%
	PET-NVIE	31%	98%		
	PET-CDRIE	72%	83%		

Abbreviations: TTE: transthoracic echocardiography, TEE: transesophageal echocardiography, CCT: cardiac computed tomography, PET: ^18^F-fluorodeoxyglucose positron emission tomography/computed tomography, NVIE: native valve infective endocarditis, PVIE: prosthetic valve infective valve endocarditis, CDRIE: cardiac device-related infective endocarditis. There are limited data on cardiac magnetic resonance.

## Data Availability

No new data were created or reported in this review article.
